# Austin District Medical Society

**Published:** 1899-10

**Authors:** J. C. Anderson, W. A. Harper

**Affiliations:** President; Secretary pro tem


### Austin District Medical Society.

The forty-seventh quarterly meeting of the Austin District Medical Society met yesterday in the Knights of Pythias Hall. Those present were: Drs. A. N. Denton, L. L. Lacy, F. E. Daniel, I. J. Jones, T. J. Bennett, C. O. Weller, H. B. Hill, T. R. Pettway and W. A. Harper, Austin; L. W. Cock, San Marcos; W. R. Blailock, McGregor; G. T. King, Elgin; A. Nowlin, J. D. Porter and — Brown, Hutto; J. S. Watson, Manor; W. E. Holtzclaw and J. A. Gillis, Buda; J. M. Witt, Bartlett; T. M. Harrell, Round Rock; D. M. Crooke, Jonah; E. M. Thomas, Georgetown; J. C. Anderson, Granger; August Kuhn, Walburg.

The meeting was called to order at 10:30 by its president, Dr. J. C. Anderson, of Granger. The secretary, Dr. S. E. Hudson, being absent, Dr. W. A. Harper was appointed secretary pro tem.

The minutes of the previous meeting being read, the program of the day was taken up.

Dr. J. M. Poyner, who was to have read a paper on "Puerperal Eclampsia," was absent.

Dr. E. M. Thomas read a paper entitled "Pernicious Anæmia." Discussion was opened by Dr. I. J. Jones, followed by Drs. Bennett, Denton, Nowlin, Pettway, Watson and Anderson. Dr. Bennett next read a paper entitled "Some Recent Cases of Appendicitis," includ-a report of six cases operated upon. [Published herewith.—Ed.]

Discussion of Dr. Bennett's paper was postponed until the afternoon.

Before adjourning for dinner, Dr. Lacy exhibited to the society an antiseptic telephone mouthpiece, which he has recently invented.

Dr. H. L. Hilgartner in the afternoon read a paper on "Preliminary Iridectomy for Senile Cataract." Paper discussed by Drs. Denton and Anderson.

The discussion of Dr. Bennett's paper, read in the forenoon, was next taken up and discussed at length by Drs. Denton, Blailock, Hill, Lacy, Jones and Anderson.

Dr. W. A. Harper followed with a paper on "Diphtheria." Dr. Harper's paper was discussed by Drs. Gillis, Watson, Pettway, Nowlin, Bennett, Hill, Kuhn and Anderson.

Drs. J. D. Porter, of Hutto, and D. M. Crooke, of Jonah, were elected to membership.

Society adjourned until next regular meeting in December.

J. C. Anderson, M. D.,

President.

W. A. Harper, M. D.,

Secretary pro tem.

---

The Eleventh Annual Meeting of the Tri-State Medical Society of Alabama, Georgia and Tennessee, will be held in Chattanooga, Tuesday, Wednesday and Thursday, October 24th, 25th and 26th, 1899.

Those desiring to read papers should send titles to Frank Trester Smith, Secretary, Chattanooga.

This meeting promises to be one of the best in the history of the society.

